# Preventing vision loss due to diabetic retinopathy

**Published:** 2023-07-07

**Authors:** Nyawira Mwangi, Covadonga Bascaran

**Affiliations:** 1 Deputy Director Academics, Kenya Medical Training College, Nairobi, Kenya.; 2 Clinical Research Fellow, London School of Hygiene and Tropical Medicine, UK.

**Links and referrals between diabetes and DR services are vital.**

**Figure:**
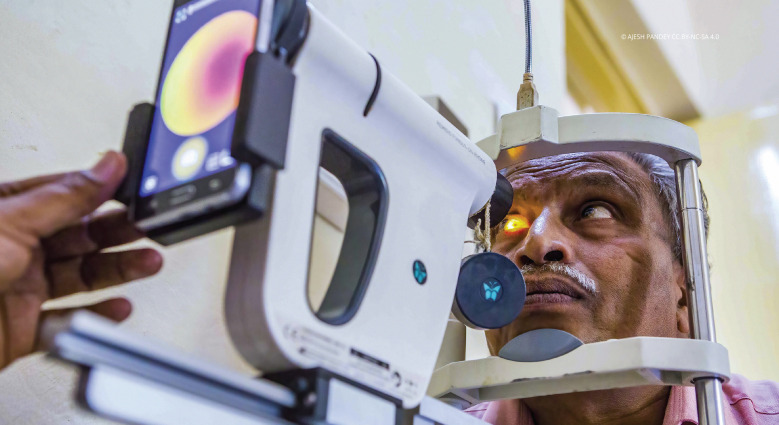
A patient is screened for signs of diabetic retinopathy. **INDIA**

Diabetic retinopathy (DR) is a non-communicable eye disease caused by diabetes mellitus; it is also a leading cause of avoidable blindness. The landscape of screening, classification, grading, diagnosis, and treatment of DR has undergone tremendous changes since the *Community Eye Health Journal’s* 2015 issue on DR. Technological advances in screening and grading, and the widespread use of anti-VEGF, are being seen in many DR programmes around the world.

## The patient’s journey

**Figure 1:**
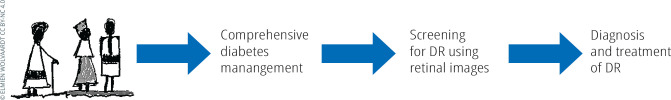
The patient’s journey.

At the centre of any DR programme, the key consideration must always be the person with diabetes and their journey towards preventing visual loss caused by DR (Figure 1).

Throughout this journey, there must be effective linkages and referral mechanisms (Figure 1, blue arrows) to ensure that people with diabetes can progress through the different stages, from diabetes management, to DR screening, to diagnosis and treatment.

### From diabetes management to retinal screening

Everyone with a confirmed diagnosis of diabetes must have their eyes screened regularly in order to identify early signs of diabetic retinopathy. This is typically done yearly, or every two years.

***How to support the patient’s journey:*** As an eye care professional, ensure that health personnel caring for diabetes patients (e.g., diabetes nurses, physicians, and/or endocrinologists) know where and when to send their patients for retinal screening. They must tell their patients about the need for regular retinal checks in order to prevent visual impairment. They should also direct the patient to the screening site, which may be at the diabetes clinic or in primary or secondary care, depending on the context.

## Sending diabetes patients for screening

Digital retinal cameras have changed the way DR screening is done and their use is now extended to many low- and middle-income settings. In this issue, we discuss the different types of cameras and the criteria to consider when choosing a camera, offer guidance on looking after the camera, provide practical tips on how to take good quality retinal images, and discuss considerations for storing and managing retinal images.

Once the retinal images have been taken, they are examined to look for signs of DR and graded according to the chosen classification system; this determines whether the person needs to be referred to the eye clinic for further examination by an ophthalmologist. Different countries and expert groups have created DR grading classification systems to suit their context, and it is a good idea to be aware of how different classifications relate to each other and to the widely used International Clinical Diabetic Retinopathy (ICDR) severity scale.

Depending on the DR programme and context, grading may be done by an ophthalmologist or a trained grader. Due to the large number of images that need to be graded, most settings are now training allied health personnel and even non-health workers to grade retinal images. Artificial intelligence technology, which has rapidly developed in the field of DR in the last decade, offers new opportunities to assist with the delivery of DR programmes through automated grading of retinal images. Many of the commercially available platforms for DR grading utilise the ICDR severity scale.

***How to support the patient’s journey:*** Once the image is taken, give the result to the patient immediately (if the person taking the images is also a trained grader) or, if the images are graded at a later date, contact the patient to share the results with them. If the patient is found to have DR, and must be referred, give them enough information about the importance of attending the eye clinic for a full examination, the risks of DR, and details about where the clinic is, and what they should expect when they attend.

## Clinical diagnosis and management

When the person attends the eye clinic, a full eye examination, final diagnosis, and management plan is put together by the ophthalmologist. Typically, treatment for sight-threatening DR consists of laser and/or anti-VEGF injections. In this issue, we provide an update on the management of sight-threatening DR, including alternative laser delivery methods and the increasingly important role of anti-VEGF, particularly in resource-constrained settings.

***How to support the patient’s journey:*** If the patient needs treatment, this must be explained in detail and the patient must consent to it. As DR treatment is delivered in more than one session, the patient must be informed of this early on, so that they can plan their attendance at the clinic.

## Developing a DR screening and treatment programme

At programme level, strategies for efficient, high-coverage screening, referral, and effective treatment remain challenging. Whilst there are effective interventions to screen and treat DR that can prevent vision loss, few countries have successfully implemented high-coverage DR programmes.

**Figure 2:**
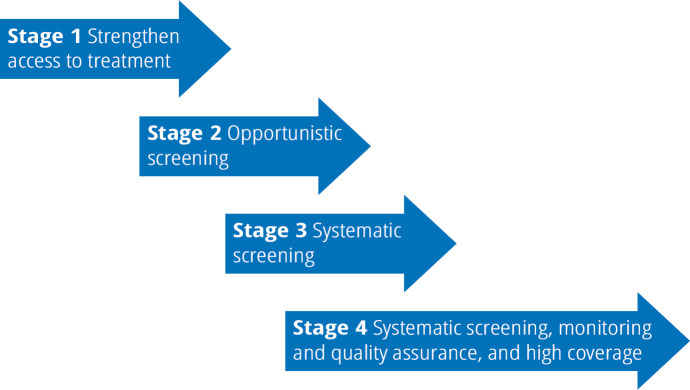
The four stages in DR programme development

DR programmes are likely to progress through different stages of development, with the ultimate goal of ensuring that all people with diabetes are screened and appropriately referred for treatment (Figure 2).

The first stage for any programme is to establish effective treatment services that are accessible and affordable to the population that they serve. These services are likely to vary in different contexts but, broadly, require the provision of laser treatment and anti-VEGF injections.

The second stage is to establish screening services for early identification of DR. This may begin as an opportunistic service, for example, screening the retina of all people with diabetes already attending a health facility for other reasons unrelated to their eyes. From there, screening may progress to being systematic, that is obtaining a register of all people diagnosed with diabetes and delivering regular screening to that population. A final stage would involve increasing the number of people that are systematically enrolled in the screening programme, aiming to achieve 80% coverage or more, and also embedding quality assurance in the programme so that the accuracy of screening can be monitored and maintained.

In summary, establishing good treatment services for DR is a priority in most countries, followed by delivering screening services for early identification of DR. Programmes must be designed with the patient’s journey in mind, so that they can progress through the clinical pathway. Both the design of the programme and the patient journey will vary from setting to setting; the context and local needs should always be taken into consideration.

